# Fulminant Course of Crohn’s Disease: A Case Report on Necrotizing Fasciitis and Septic Shock as Lethal Complications

**DOI:** 10.7759/cureus.87783

**Published:** 2025-07-12

**Authors:** Sergej Marjanovic, Sonja Dukic, Jovana Stanisavljevic

**Affiliations:** 1 Department of Anesthesiology, Resuscitation and Critical Care, University Clinical Centre of Serbia, University of Belgrade, Faculty of Medicine, Belgrade, SRB

**Keywords:** crohn's disease, intensive care, necrotizing fasciitis, septic shock, soft tissue infection

## Abstract

Necrotizing fasciitis (NF) is a rare but potentially fatal soft tissue infection that presents a particular challenge in immunocompromised individuals, including patients with Crohn's disease (CD). Although perianal manifestations are common in CD, progression to NF remains exceedingly rare. We report the case of a 39-year-old man with CD, complicated by perianal involvement, multiple surgical interventions, and a loop colostomy. The patient presented with right lower extremity pain and edema, with imaging indicating extensive subcutaneous and fascial inflammation consistent with NF. Emergent surgical debridement and broad-spectrum antibiotic therapy were initiated; however, despite intensive care management and escalation of antimicrobial therapy, the patient’s condition continued to deteriorate. Cultures identified a polymicrobial infection including vancomycin- and linezolid-resistant enterococci (VLRE). The patient ultimately succumbed to septic shock. This case highlights the potential for atypical and fatal infectious complications in patients with long-standing CD, particularly those undergoing immunosuppressive therapy and repeated surgical interventions. Timely diagnosis and aggressive management are crucial for improving outcomes in NF; however, treatment is often complicated by diagnostic delays, rapid clinical deterioration, and the emergence of multidrug-resistant organisms, all of which pose significant therapeutic challenges.

## Introduction

Crohn's disease (CD) is a type of inflammatory bowel disease (IBD), similar to ulcerative colitis. The etiology of CD is unknown, and it is associated with many pathophysiological mechanisms, primarily of an immunological nature [1]. This condition can involve any part of the gastrointestinal tract [1]. The main symptoms of CD include fatigue, fever, abdominal pain and cramping, diarrhea, weight loss, and the presence of strictures, fistulas (perianal, enteroenteric, enterocolic, enterovesical, enterocutaneous, colocutaneous, etc.), and abscesses, often leading to complications such as bowel obstruction [2]. CD is a multisystem disease and can present with a wide range of extraintestinal manifestations such as sacroiliitis, enteropathic arthritis, episcleritis, primary sclerosing cholangitis, and others [1,2].

Necrotizing fasciitis (NF) is a life-threatening, severe form of skin and soft tissue infection, characterized by rapidly progressing necrosis of the muscle fascia and subcutaneous adipose tissue [3]. Early diagnosis and prompt initiation of therapy are extremely important and should include the administration of empiric broad-spectrum antibiotics, along with aggressive surgical procedures for abscess drainage and debridement of the infected site [3].

Septic shock represents the most severe manifestation of sepsis, characterized by profound circulatory and cellular/metabolic dysfunction, often leading to organ failure and high mortality. The growing prevalence of multidrug-resistant (MDR) pathogens further complicates treatment and underscores the urgency of early recognition and targeted antimicrobial therapy [4].

This article presents a rare and devastating case of a patient with Crohn's disease complicated by rapidly progressive and fatal right lower extremity necrotizing fasciitis despite aggressive management in the intensive care unit.

## Case presentation

A 39-year-old man with a 15-year history of diagnosed CD presented with a 3-month history of progressively worsening chronic pain and swelling of the right lower extremity. Fifteen years ago, the patient underwent right hemicolectomy with primary anastomosis due to colonic perforation. Thereafter, the diagnosis of CD was made based on histopathological findings. Infliximab therapy was initiated (5 mg/kg IV at weeks 0, 2, and 6, followed by every 8 weeks). Eight years later, the patient developed a complex perianal fistula, marking the beginning of a prolonged course of perianal CD. Since then, the patient underwent surgical interventions on nine occasions due to recurrent CD-related complications. Because of inadequate disease control, a loop colostomy was performed approximately 18 months prior to admission. The patient was a heavy smoker with several unsuccessful attempts to quit. Upon admission, the patient reported low back pain and intermittent drainage from a perianal fistula. Notably, two months prior to admission, the patient experienced similar painful symptoms, which at that time were attributed to an interpolar right renal calculus, confirmed by ultrasonography in the local city hospital. Upon hospital admission, the patient presented with the following signs: heart rate of 110 beats per minute, without abnormal lung sounds, oxygen saturation of 97%, and was afebrile. Physical examination revealed mild tenderness to palpation, erythema, edema, and increased warmth of the right lower extremity. The patient was underweight, with a body mass index (BMI) of 16.5 kg/m². Laboratory analyses showed a positive inflammatory syndrome, characterized by elevated white blood cell (WBC) count, absolute neutrophil count, platelets, C-reactive protein (CRP), and fibrinogen, along with stable anemia of chronic disease (Table 1).

**Table 1:** Admission laboratory findings demonstrating a positive inflammatory syndrome, characterized by elevated white blood cell (WBC) count, absolute neutrophil count, C-reactive protein (CRP), platelet count, and fibrinogen levels L – liter; mg/L – milligrams per liter; g/L – grams per liter; g/dL – grams per deciliter

Lab Test	Result	Reference range
White blood cells (WBC)	16 × 10⁹/L	4.2 – 11.5 × 10⁹/L
Neutrophils	12 × 10⁹/L	1.5 – 7.5 × 10⁹/L
Platelets	600 × 10⁹/L	120 – 450 × 10⁹/L
C-reactive protein (CRP)	40 mg/L	0.0 – 5.0 mg/L
Fibrinogen	7 g/L	2.0 – 4.0 g/L
Hemoglobin	10.5 g/dL	12.5 – 17.5 g/dL

**Figure 1:**
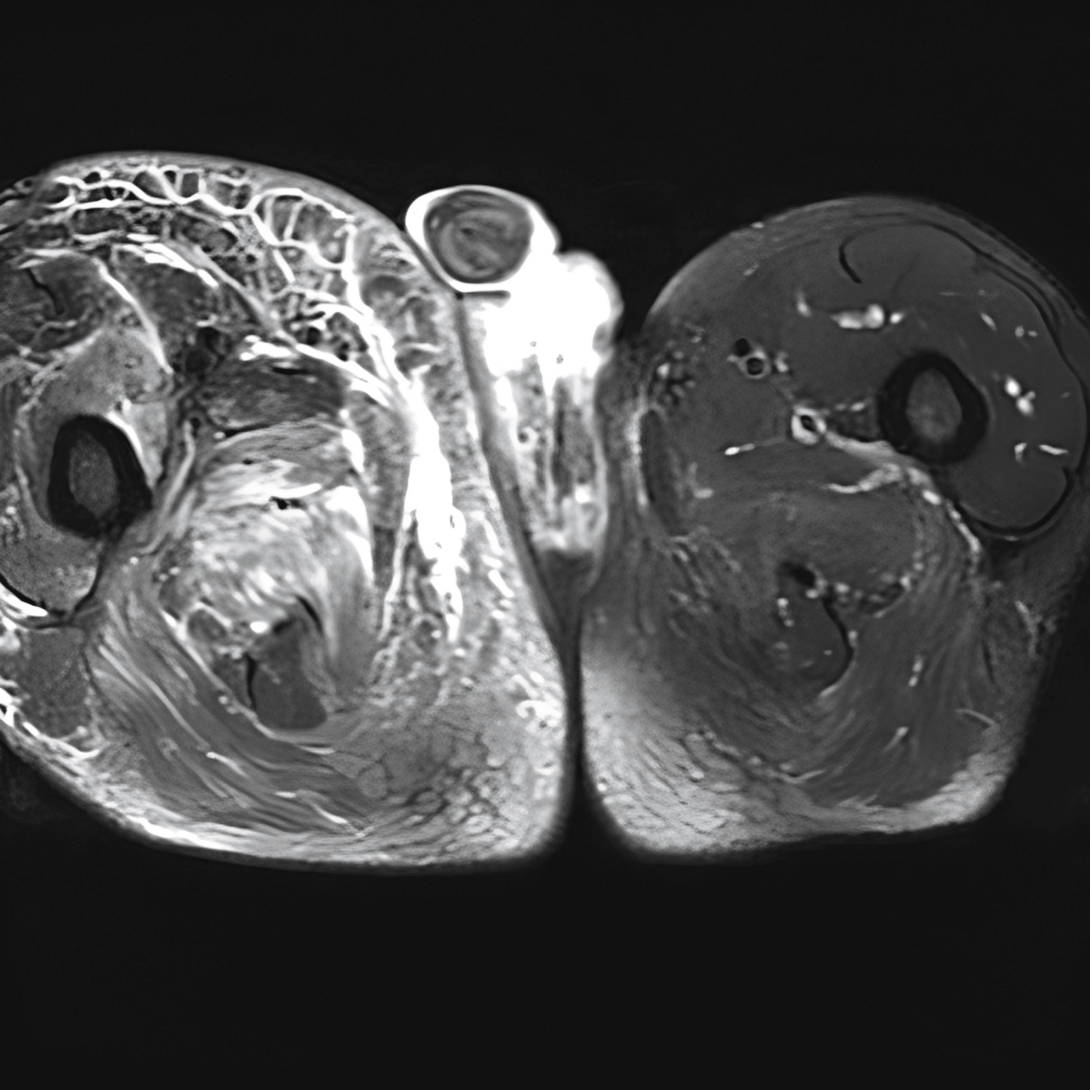
Axial T2-weighted fat-suppressed MRI of the thighs demonstrating extensive hyperintense signal in the subcutaneous and deep soft tissue of the right thigh, consistent with edema and inflammation. There is associated fascial thickening. Note the asymmetry between the left and right thighs, with diffuse tissue swelling in the affected limb.

**Figure 2:**
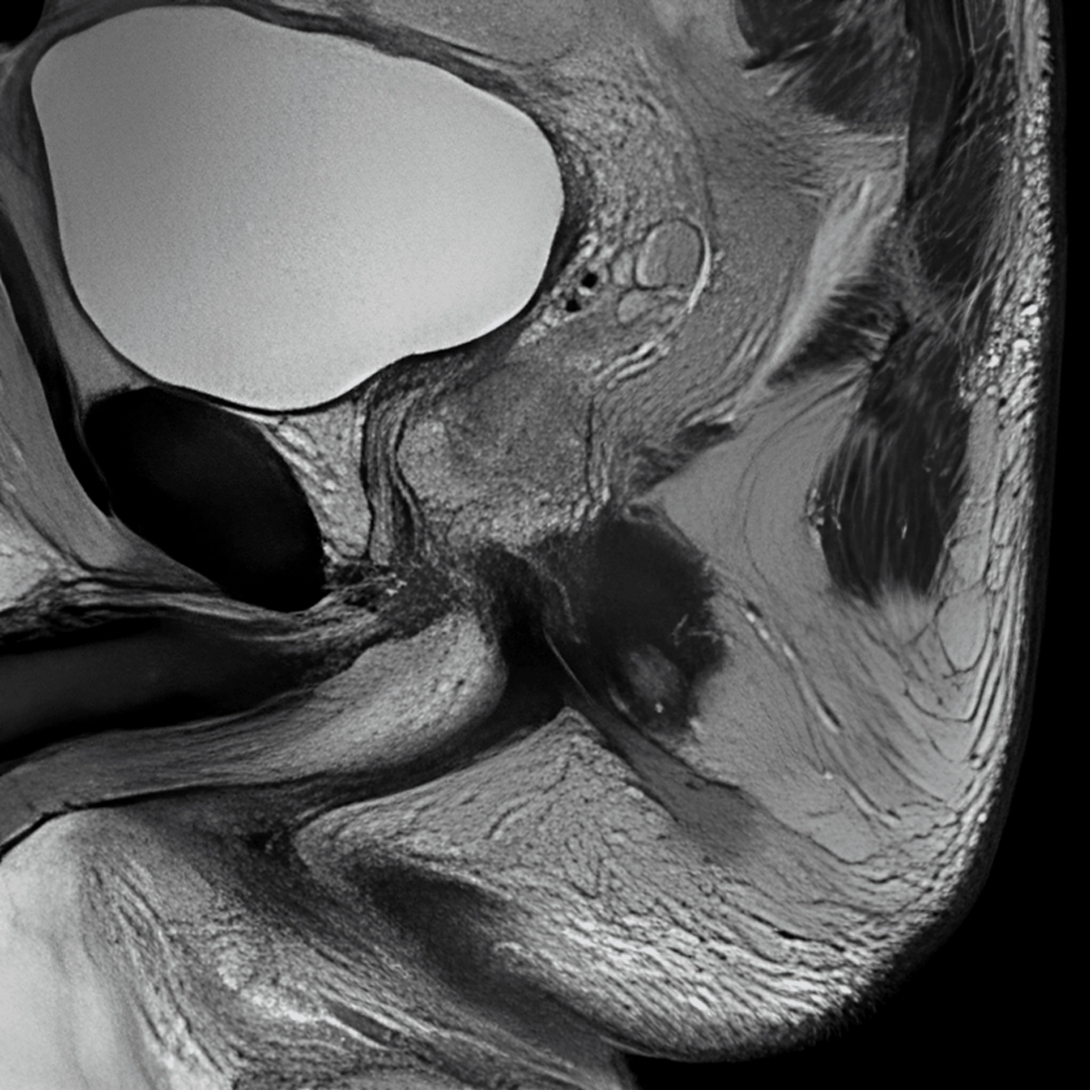
Sagittal T2-weighted MRI of the pelvis demonstrating a hyperintense tract consistent with a fistulous pathway

Colour Doppler ultrasound revealed evidence of venous thrombosis in the right inguinal region, and therapy with fraxiparine (0.6 mL SC twice daily) was initiated. Colonoscopy was unremarkable. On the second day of hospitalization, magnetic resonance imaging (MRI) of the abdomen and pelvis was performed to evaluate a potential intra-abdominal or pelvic source of infection. The scan revealed extensive subfascial and interfascial fluid collections, along with thickening and hyperintense signal of the muscle fascia on T2-weighted images, consistent with fascial inflammation. These findings, particularly the deep intramuscular fascial involvement and edema, are characteristic of NF, even in the absence of gas formation or hypoenhancement due to necrosis (Figure 1). Additionally, MRI demonstrated complex transsphincteric fistulas with an internal opening in the anterior wall of the anal canal and an associated abscess cavity. The abscess communicated with a second fistulous tract, which opened posterior to the scrotum and terminated blindly near the coccyx (Figure 2). A cutaneous opening was also noted along this tract, suggestive of ongoing drainage. The diagnosis of NF was made, and urgent surgical treatment was initiated. During surgery, extensive necrotic tissue was identified in the subcutaneous and fascial planes of the right lower extremity. Surgical debridement included excision of devitalized skin, subcutaneous fat, and necrotic fascia, with preservation of viable muscle tissue. Purulent material was drained, and deep fascial layers were thoroughly irrigated. Wound edges were left open for ongoing assessment and daily dressing changes.

**Figure 3:**
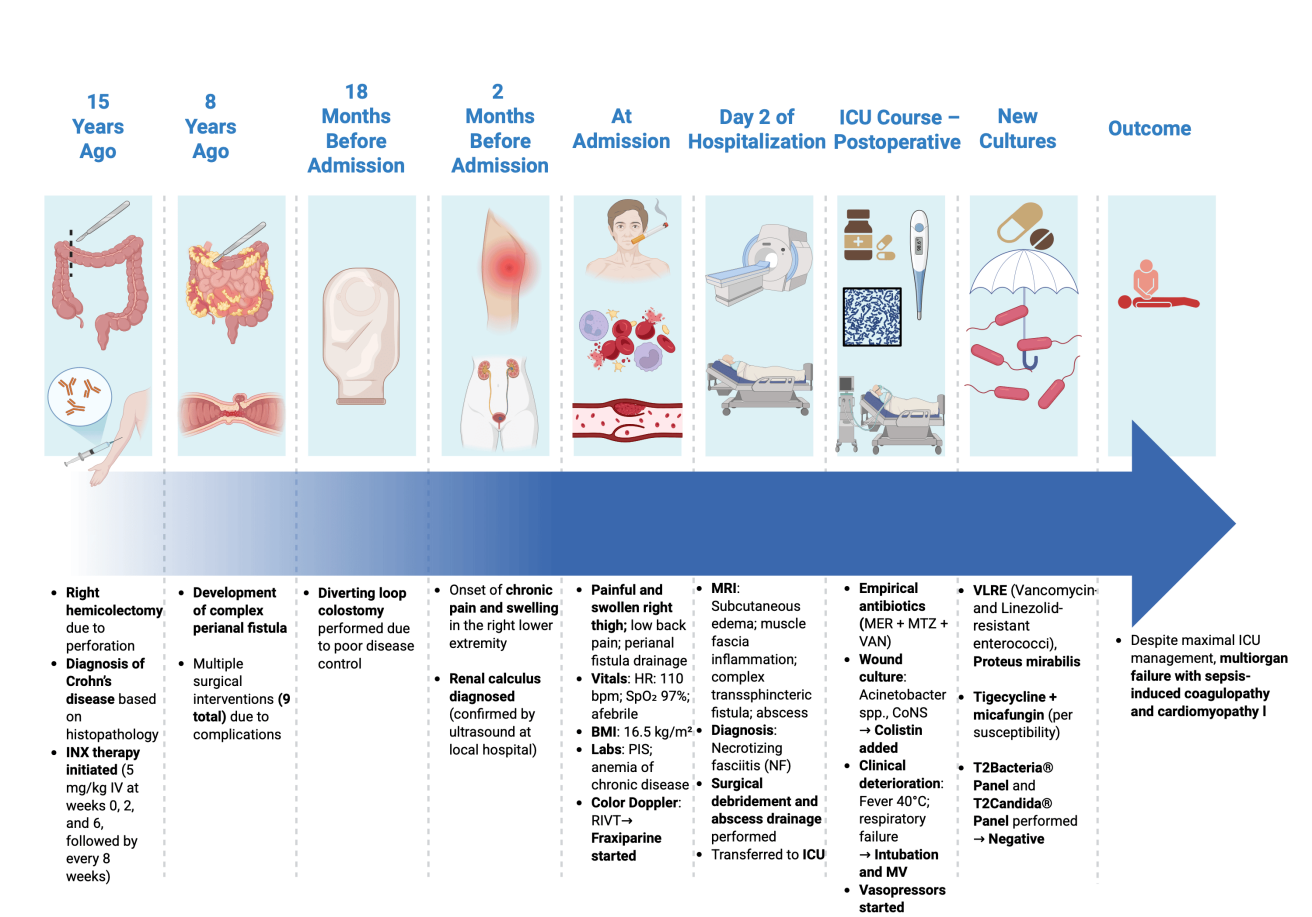
Patient timeline highlighting major clinical events INX – infliximab; HR – heart rate; bpm – beats per minute; BMI – body mass index; PIS – positive inflammatory syndrome; RIVT – right inguinal vein thrombosis; MRI – magnetic resonance imaging; ICU – intensive care unit; MER – meropenem; MTZ – metronidazole; VAN – vancomycin; CoNS – coagulase-negative staphylococci; MV – mechanical ventilation Image Credits: Created by Sergej Marjanovic using BioRender.

After surgery, the patient was transferred to the Intensive Care Unit (ICU), which represents a tertiary-level intensive care facility in the country. Postoperative wound management was conducted on a daily basis within the ICU, in accordance with aseptic protocols. The patient was treated immediately empirically with broad-spectrum antibiotics, including meropenem (1 g IV three times daily), metronidazole (500 mg IV three times daily), and vancomycin (2 g IV twice daily). The patient remained subfebrile (measured at 37.5° C) during the first postoperative day. On postoperative day 3, cultures from the wound were positive for Acinetobacter spp. and coagulase-negative Staphylococcus. Antibiotic therapy was adjusted according to the antibiogram, and colistin (loading dose: 9 million IU IV, followed by 4.5 million IU IV twice daily) was added to the previous three-antibiotic regimen. However, there was no clinical or laboratory improvement, and the patient became febrile (up to 40°C) on postoperative day 5. Due to worsening respiratory status, the patient was intubated and connected to invasive mechanical ventilation. To achieve and maintain adequate perfusion, norepinephrine infusion was initiated, followed by the addition of vasopressin and hydrocortisone. Additional microorganisms, including Enterococcus spp. and Proteus mirabilis, were later isolated from wound cultures on postoperative day 7. The Enterococcus spp. isolates included vancomycin- and linezolid-resistant enterococci (VLRE). Tigecycline (100 mg IV twice daily) was subsequently added based on antibiogram results, along with micafungin (100 mg IV once daily). Due to the severity of the clinical picture, culture-independent assays - T2Bacteria®Panel and T2Candida®Panel - were performed in order to detect potential bloodstream pathogens. Both results returned negative. Despite the implementation of all therapeutic measures in the ICU, including advanced antimicrobial therapy, multiorgan support systems, and adherence to sepsis management protocols, the patient developed sepsis-induced coagulopathy and cardiomyopathy. These complications delayed and ultimately precluded the performance of a second-look surgical debridement. The patient's condition continued to deteriorate, and he passed away on postoperative day 10 (Figure 3).

## Discussion

NF is a serious, rapidly progressive infection of the soft tissue, whose main characteristic is necrosis of the muscle fascia and subcutaneous adipose tissue. The most frequent localization of infection includes the lower extremities, perineum, and abdomen [4,5].

There are only a few case reports of NF in patients with CD in which NF may present either as the first manifestation of disease or during its course and progression. The mortality rate is generally high and varies across different studies, ranging from 19% to 76% [3,5]. An immunocompromised status in patients with NF can be a potential risk factor for high in-hospital mortality and for delay in the initial surgical intervention [6]. Our patient was on biological therapy with the anti-TNF monoclonal antibody infliximab, which has been shown to more frequently lead to NF in patients with CD [7].

Wong and Wang classified NF into three different clinical stages - early, intermediate, and late - based on clinical features [8]. In the early stage, NF may share overlapping features with other severe soft tissue infections, such as cellulitis and erysipelas, presenting only with tenderness to palpation, erythema, swelling, and warmth of the skin. According to the Laboratory Risk Indicator for Necrotizing Infection Score (LRINEC), our patient had a score of eight, which is associated with a high risk of developing NF. It evaluates different laboratory parameters, such as C-reactive protein, white blood cell count, hemoglobin concentration, serum sodium, creatinine, and blood glucose levels (Table 2). LRINEC is a simple and accessible scoring system that helps distinguish between NF and other soft tissue infections [3,9].

**Table 2:** The patient’s LRINEC score is presented with each laboratory parameter listed alongside its scoring criteria, measured value, and assigned points. A total score of ≥ 8 indicates a high risk for necrotizing fasciitis. LRINEC – Laboratory Risk Indicator for Necrotizing Fasciitis

Parameter	Scoring Criteria	Patient Value	Points
C-reactive protein (mg/L)	0 points: < 150	40	0
	4 points: ≥ 150		
White blood cell count (×10⁹/L)	0 points: < 15	16	1
	1 point: 15 - 25		
	2 points: > 25		
Hemoglobin (g/dL)	0 points: > 13.5	10.5	2
	1 point: 11 - 13.5		
	2 points: < 11		
Sodium (mmol/L)	0 points: ≥ 135	131	2
	2 points: < 135		
Creatinine (mol/L)	0 points: ≤ 141	145	2
	2 points: > 141		
Glucose (mol/L)	0 points: ≤ 10	12.2	1
	1 point: > 10		
Total Score: 8			

Second-look surgery is widely recognized as a critical component in the management of necrotizing fasciitis (NF), as it allows for the timely identification and removal of any residual necrotic tissue, thereby reducing the infectious burden and facilitating hemodynamic stabilization. Repeated surgical debridement has been shown to significantly improve outcomes by enhancing source control in severe soft tissue infections [3]. In this case, a second-look surgery was initially planned as part of the overall management strategy. However, the procedure could not be performed due to the patient’s abrupt and progressive clinical deterioration. Despite intensive care support, the patient developed sepsis-induced coagulopathy and cardiomyopathy, progressive multiorgan failure, and was severely malnourished - all of which substantially increased the perioperative risk and ultimately rendered the patient unfit for reoperation.

Patients with CD who develop NF have increased energy requirements as a result of both the septic and surgical burden, as well as the metabolic impact of the underlying inflammatory disease [10,11]. According to the Global Leadership Initiative on Malnutrition (GLIM), a diagnosis of malnutrition can be established based on one phenotypic criterion (low body mass index) and one etiologic criterion (inflammation) [12]. The patient’s energy requirements were assessed using indirect calorimetry (IC), the gold standard for measuring patient resting energy expenditure (REE) in clinical practice. Therefore, the nutrition therapy of our patient was optimized, and early enteral nutrition (EEN) was started within 72 hours postoperatively, in combination with supplemental parenteral nutrition (PN), including immunonutrition to meet energy and protein requirements.

Perianal manifestation occurs in approximately 20-54% of patients with CD [13]. Regardless of the type of fistulas, more than 90% of patients with CD will require multiple surgical procedures [13]. Due to the presence of a complex perianal fistula in our patient, we strongly suspect that the NF developed as a result of direct/local spread of infection. Additionally, immunosuppression and malnutrition were significant contributing risk factors that facilitated the development of this severe soft tissue infection.

The detection of vancomycin- and linezolid-resistant enterococci (VLRE) in this case is of particular clinical relevance, as such strains remain exceedingly rare. According to a review summarizing data from two studies monitoring linezolid susceptibility, the overall prevalence of linezolid resistance among clinical Enterococcus spp. isolates remain low (<1%) [14]. Immunodeficiency has been recognized as one of the possible risk factors for the presence of VLRE in clinical specimens, and our patient indeed fulfilled this criterion based on several factors outlined earlier in this case report. This complicates treatment and requires strict infection control measures (e.g., isolation precautions and contact-based protocols) to prevent nosocomial transmission, as well as the implementation of effective antimicrobial stewardship strategies. Early identification and close collaboration with infectious disease specialists are essential to improve outcomes [15].

NF is associated with septic shock [9]. On postoperative day 10, our patient became hemodynamically unstable. In accordance with the Surviving Sepsis Campaign (SSC) guidelines for sepsis and septic shock, norepinephrine was initiated (0.25-0.5 μg/kg/min) as first line therapy. Subsequently, vasopressin (0.01-0.03 U/min) and hydrocortisone (200 mg/day as continuous IV infusion) were added due to refractory septic shock. In immunocompromised patients presenting with septic shock, as seen in this case, rapid clinical deterioration is common. Therefore, early recognition, prompt fluid resuscitation, empirical antibiotic administration within the first hour, obtaining cultures with sensitivity testing, and urgent source control are critical components of management to improve patient outcomes [16].

Although there were no clear indications for continuous renal replacement therapy (CRRT), given the preserved renal function throughout the course of the treatment, the potential benefit of extracorporeal blood purification techniques aimed at modulating the systemic inflammatory response, such as hemoadsorption (e.g., CytoSorb®), remains an open question. Despite the availability of such therapies at the time, they were not employed due to the absence of clear indications for initiating CRRT as the platform for hemoadsorption.

## Conclusions

This case underscores the critical importance of early recognition and timely management of NF in immunocompromised and malnourished patients with CD, particularly those with complex perianal involvement, highlighting the diagnostic challenges posed by this rare but severe extraintestinal complication. The complexity of treatment necessitates a coordinated multidisciplinary effort involving intensivists, surgeons, anesthesiologists, infectious disease specialists, and other healthcare professionals to ensure comprehensive patient care. Awareness and vigilance are paramount, as delayed diagnosis can lead to rapid deterioration and poor outcomes. Prompt surgical intervention, targeted antimicrobial therapy, intensive care support, and individualized nutritional strategies are crucial for improving prognosis. Furthermore, this case highlights the growing clinical challenge posed by multidrug-resistant pathogens, which complicate treatment strategies and may contribute to therapeutic failure. Ultimately, this case serves as a reminder to clinicians to maintain a high index of suspicion for NF in CD patients and adopt a proactive, collaborative approach to optimize clinical outcomes.
